# Implementing the human right to science in the regulatory governance of artificial intelligence in healthcare

**DOI:** 10.1093/jlb/lsad026

**Published:** 2023-10-14

**Authors:** Calvin W L Ho

**Affiliations:** Faculty of Law and Centre for Medical Ethics and Law, The University of Hong Kong, Cheng Yu Tung Tower, Centennial Campus, Pokfulam, Hong Kong

**Keywords:** Right to Science, Artificial Intelligence, Medical Device, governance, software

## Abstract

Artificial intelligence (AI) enables a medical device to optimize its performance through machine learning (ML), including the ability to learn from past experiences. In healthcare, ML is currently applied within controlled settings in devices to diagnose conditions like diabetic retinopathy without clinician input, for instance. In order to allow AI-based medical devices (AIMDs) to adapt actively to its data environment through ML, the current risk-based regulatory approaches are inadequate in facilitating this technological progression. Recent and innovative regulatory changes introduced to regulate AIMDs as a software, or ‘software as a medical device’ (SaMD), and the adoption of a total device/product-specific lifecycle approach (rather than one that is point-in-time) reflect a shift away from the strictly risk-based approach to one that is more collaborative and participatory in nature, and anticipatory in character. These features are better explained by a rights-based approach and consistent with the human right to science (HRS). With reference to the recent explication of the normative content of HRS by the Committee on Economic, Social and Cultural Rights of the United Nations, this paper explains why a rights-based approach that is centred on HRS could be a more effective response to the regulatory challenges posed by AIMDs. The paper also considers how such a rights-based approach could be implemented in the form of a regulatory network that draws on a ‘common fund of knowledges’ to formulate anticipatory responses to adaptive AIMDs. In essence, the HRS provides both the mandate and the obligation for states to ensure that regulatory governance of high connectivity AIMDs become increasingly collaborative and participatory in approach and pluralistic in substance.

## I. INTRODUCTION

In April 2018, the Food and Drug Administration (FDA) in the USA authorized IDx-DR (recently renamed as LumeneticsCore)^1^ to be marketed as the first Artificial Intelligence (AI) diagnostic system that does not require clinician interpretation to detect greater than a mild level of diabetic retinopathy in adults diagnosed with diabetes.^2^ In essence, this fully autonomous prescription software device incorporates an adaptive algorithm to evaluate images of the eye taken with a retinal camera that are uploaded to a cloud server.^3^ A screening decision is made by the device as to whether the individual concerned is detected with ‘more than mild diabetic retinopathy’. If so, this individual is referred to an eye care professional for medical attention. From a regulatory standpoint, software intended for use by healthcare professionals and patients in the USA must comply with basic regulatory requirements, including medical device listing and labeling, premarket notification (unless exempted) or premarket approval (PMA), investigational device exemption for clinical studies, and quality system regulation. In Europe, IDx-DR has secured a Class IIa CE mark (further discussed below) for the autonomous detection of referable diabetic retinopathy.^4^ Similar AI-based medical devices (AIMDs) for the remote diagnosis of diabetic retinopathy developed by Shenzhen Guiji and Shanghai Yingtong have received regulatory approval from the National Medical Products Administration (NMPA),^5^ which is responsible for regulating medical devices (whether standalone AIMDs or otherwise) in China.^6^

Like conventional medical devices,^7^ the regulation of AIMDs is risk-based, context-specific, and case-sensitive. Unlike the former however, the risk profiles of technically sophisticated AIMDs can significantly deviate from the versions that secured regulatory approval since some of these devices have the capability of adapting to new conditions through ‘unsupervised’ learning, and thereby present concerns over patient safety and effectiveness. Recognizing that the conventional point-in-time regulatory approach is not well suited for AIMDs that undergo fast-paced cycles of iterative modifications, regulators from major AIMD jurisdictions have, working singly and collaboratively through the International Medical Device Regulators Forum (IMDRF), identified regulatory principles and mechanisms that facilitate risk categorization and management, but these endeavors are still ongoing.^8^ The FDA has, for instance, presented a proposed regulatory framework for certain types of anticipated software modifications and associated methodology used to implement the changes in a discussion paper.^9^ This proposed framework was developed based on FDA’s existing risk management principles, benefit–risk framework, and organization-based total product lifecycle approach, as well as the IMDRF’s risk categorization principles, and forms the basis of an action plan for AI or machine learning (ML) software as a medical device following extensive feedback received from its stakeholders.^10^ This regulatory framework proposed by the FDA will be further discussed below. For now, it suffices to say that the emerging form of regulatory governance for AIMDs is decentralized, collaborative, and pre-emptive.^11^

This paper examines how the human right to science (HRS) may be applied to explain a shift from the point-in-time regulation of non-AI medical devices to the total device/product lifecycle regulation of AIMDs like IDx-DR. Additionally, it explores how HRS could help steer regulatory evolution toward the establishment of a regulatory approach that is participatory and, in terms of its epistemic (knowledge) features, pluralistic and anticipatory. This approach in turn helps to sustain and stabilize a sociotechnical imagination of a desirable future that is ‘animated by shared understandings of forms of social life and social order attainable through, and supportive of, advances in science and technology’ and consistent with a rights-based approach in general.^12^

Looking beyond the narrow construct of risks that has been foundational to the regulation of non-AI medical devices,^13^ this paper argues that HRS could serve as a cultural prism that magnifies specific contexts of rationalization. By making explicit the social conditions, moral commitments, political movements, institutional arrangements, and technical means that underscore the regulatory governance of non-AI medical device, I explain why a rights-based approach is not only necessary for its focus on participation and collaboration,^14^ but is further consistent with the wider initiative of developing learning healthcare systems.^15^ After all, the language of ‘risks’ offers but one (albeit crucial) avenue to consider metaphors (like ‘technical’, ‘ethical’, and ‘legal’) which serve to frame fundamental conceptions of ‘regulatory space’ and ‘governance’, as well as how they are being put together. The high connectivity of AIMDs and their capacity to adapt to their digital environment in order to optimize performance underscore the need to re-configure our understanding of contexts and meanings. Active regulatory participation cannot be limited to the pilot or feasibility stages of medical device trials. If continuous risk-monitoring is required to support the use of AIMDs in a learning healthcare system, more robust, responsive, and participatory regulatory mechanisms are needed, not less.^16^ There is of course a danger that the total device/product lifecycle regulatory approach could give rise to intrusive political constraints and excessive bureaucratic controls on scientific activities. Here too, the HRS provides instructive guidance in that enjoyment of the benefits of science is in many ways contingent on existence of effective protection of the freedom that is indispensable for scientific research and creative activity, as well as the moral and material interests resulting from any scientific production. Additionally, overly intrusive regulatory intervention will not be consistent with the obligation that states have under the HRS to advance science and to protect and disseminate scientific knowledge and its applications. With a growing number of AIMDs being developed and introduced into clinical care, conventional thinking of medical device regulation that is rooted in the binary of risk assessment and risk management (or otherwise framed in terms of safety and effectiveness) is no longer sustainable. AIMDs provide the opportunity to re-conceptualize the nature and goals of medical devices regulation by drawing reference to HRS, particularly in its participatory or governance sense,^17^ as well as to other human rights that may be linked to it.

While the focus of this paper will be mainly on the regulatory approach of the FDA and its implementation of the principles of the IMDRF, references will be made to regulatory developments in China and in the European Union (EU) for the purpose of illustration rather than comparison. This may be useful to reveal ingrained normative commitments that distinguish political communities, particularly in their ways of knowing and reasoning.^18^ Additionally, the term ‘regulation’ in this paper is applied broadly as comprising any instrument (legal or non-legal in character) that is designed to channel group behavior.^19^ Regulatory governance rather than simply regulation is used to highlight the increasingly ‘decentered’ nature of intentional attempts to manage risk that are undertaken not only by state actors, but also by non-state actors, including commercial firms and civil society organizations. Where technological innovation is concerned, concerns about ‘risks’ and the need to tame them have been central to regulatory governance.^20^ Here, regulatory response is taken to reduce the uncertainty and instability of risk conception and mitigation measures, by directing or influencing actors’ behavior to accord with socially accepted norms and/or to promote desirable social outcomes.^21^

There are five main sections to this paper. In Section II that follows, I first explain the normative content of HRS and the core obligations that it imposes on regulators. One such obligation is for regulators to prevent or mitigate risks that arise from technological development and use. I then examine how risk-based approaches have been implemented in major AIMD jurisdictions like the USA, the EU, and China to prevent or mitigate risks that arise from the use of medical devices in Section III. However, these risk-based approaches are unlikely to be effective for AIMDs with ML capability for the reasons that I set out in Section IV. Apart from technological developments that limit regulatory effectiveness, risk-based approaches do not adequately account for the right of every person to participate in and to enjoy the benefits of scientific and technological progress. Recent changes introduced to regulate AIMDs as ‘software as medical device’, along with the introduction of associated regulatory mechanisms, seek to address some of the limitations. In Section V, I explain that while these developments attempt to improve regulatory effectiveness through means that enhance participation and collaboration, they do not go far enough. I then put forward, in Section VI, how a rights-based approach that is centered on HRS could be a more effective response to the regulatory challenges posed by AIMDs.

## II. HUMAN RIGHT TO SCIENCE

The HRS is set out as the key source of specific rights and freedoms to which all humans are entitled in relation to scientific progress and its applications under Article 27 of the Universal Declaration of Human Rights (UDHR),^22^ and Article 15 of the International Covenant on Economic, Social and Cultural Rights (ICESCR).^23^ In 1985, the Committee on Economic, Social and Cultural Rights of the United Nations (UNCESCR) was established to implement the ICESCR. Since then, the UNCESCR has continuously clarified the nature of the economic, social, and cultural rights (ESCR) and their justiciability in terms of the principles, standards, and procedural rules that apply. For the purposes of this paper, the UNCESCR’s articulation of the core (or minimum) content of HRS and the core (or minimum) obligations of states in this connection is most pertinent.^24^ As it explains, the following features must be present in order for HRS to enable everyone to (i) enjoy the benefits of scientific progress and it application; (ii) participate in science as part of cultural (or creative) life (hence inclusive of ‘citizen science’); (iii) benefit from the protection of the moral and material interests resulting from any scientific, literary, or artistic production for the inventor or author; (iv) enjoy freedom indispensable for scientific research and creative activity; and (v) take steps for the conservation, development, and diffusion of science.

Where emerging medical technologies are concerned, scholars explain that HRS has at least three essential rights in implementation: first, the right of everyone to benefit from and contribute to scientific and technological progress (or HRS in the public interest sense); second, the right of scientists, for instance, to do research and push forward science and technology (or HRS in a technical sense); and third, countries’ duty to provide an enabling environment (or HRS in a governance sense).^25^ The ‘benefits’ to which HRS relates is not limited to the material results of scientific research (eg an AIMD) or to the scientific knowledge and information derived from the research, but also to the role of science in forming critical and responsible citizens who are able to participate fully in a democratic society.^26^ This paper focuses on the core content and obligations of HRS in its governance sense, or what has been described as the ‘right for people to have a legislative and policy framework adopted and implemented which aims at making the benefits of scientific progress available and accessible—both through encouraging new scientific discoveries and through removing barriers for existing scientific knowledge to be used for public benefit’.^27^ In this connection, the UNCESCR indicates that states have to implement, as a matter of priority, core obligations that include^28^ (a) developing a participatory national framework law on HRS (with specification of legal remedies in case of violations) and implementing a participatory national strategy or action plan for the realization of HRS; (b) ensuring access to those applications of scientific progress that are critical to the enjoyment of the right to health and other ESCR; (c) ensuring that (in allocation of public resources) priority is given to research in areas where there is the greatest need for scientific progress in health and the well-being of the population, especially with regards to vulnerable and marginalized groups; (d) ensuing that health professionals are properly trained in using and applying modern technologies resulting from scientific progress; and (e) fostering the development of international contacts and cooperation in the scientific field. While every reasonable effort must be made to implement the core obligations of HRS using the maximum of its available resources (individually and through international assistance and cooperation), the UNCESCR reminds all states that they are also to take into account the *totality of the rights* enshrined in the ICESCR.^29^ The right to participate in and to enjoy the benefits of scientific progress in the HRS may constitute an essential tool for the realization of other ESCR.^30^

The UNCESCR’s discussion of the link between HRS and the right to health is especially instructive for its illustration of a rights-based approach to the governance of medical products.^31^ For medical technologies in general, states have a duty to make available and accessible to all persons (and especially to the most vulnerable) all the best available applications of scientific progress necessary to enjoy the highest attainable standard of health.^32^ It follows that medical devices that are safe and effective should be prioritized in national health plans in order to make the best use of available resources for the fulfillment of ESCRs. There is also an obligation for states to promote scientific research, to create new medical applications and make them accessible and affordable to everyone, especially the most vulnerable.^33^ Where some scientific research carries health-related risks, states are required to present or mitigate these risks through careful application of the precautionary principle and the protection of participants in scientific research. More specifically, states should make every effort to ensure that ‘medical treatments…are evidence-based, and that the risks involved have been properly evaluated and communicated in a clear and transparent manner, so that patients can provide properly informed consent.’^34^ In the next section, I will show that this aspect of a rights-based approach applies to non-AI medical devices that are currently in use.

## III. RISK-BASED REGULATION

In jurisdictions like the USA, the EU, and China, an essentially risk-based approach is applied to regulate medical devices. By this approach, the risk profile of these devices are regarded as quantifiable and static, in the sense that their functions are stable and their outcomes are relatively clear and predictable for the indicated uses. In the USA, the Federal Food Drug & Cosmetics Act (FD&C Act) defines the term ‘device’ as^35^

an instrument, apparatus, implement, machine, contrivance, implant, in vitro reagent, or other similar or related article, including any component, part, or accessory, which is….(2) intended for use in the diagnosis of disease or other conditions, or in the cure, mitigation, treatment, or prevention of disease, in man or other animals; or (3) intended to affect the structure or any function of the body of man or other animals, and which does not achieve its primary intended purposes through chemical action within or on the body of man or other animals and which is not dependent upon being metabolized for the achievement of its primary intended purposes.

This definition is broadly similar to a definition developed by the Global Harmonization Task Force,^36^ and adopted by the World Health Organization.^37^ For regulatory purposes, medical devices are classified based on their intended use and indications for use, degree of invasiveness, duration of use, and the risks and potential harms associated with their use. At the classification stage, a manufacturer is not expected to have gathered sufficient data to demonstrate that its proposed product meets the applicable marketing authorization standard (eg data demonstrating effectiveness).

### III.A. United States

It follows that the focus of FDA’s classification analysis is on how the product is expected to achieve its primary intended purposes.^38^ Approximately 1700 different generic types of devices have been classified by the FDA and grouped into 16 medical specialties referred to as panels.^39^ Each of these generic types of devices is assigned to one of three regulatory classes based on the level of control necessary to assure the safety and effectiveness of the device.^40^ Unless exempted,^41^ the class to which the device is assigned determines, among other things, the type of premarketing submission or application required for FDA clearance to market (a) the 510(k) pre-market notification or clearance; (b) de novo classification; or (c) PMA.

All classes of devices are subject to General Controls,^42^ which are the baseline requirements of the FD&C Act that applies to all medical devices. Special Controls are regulatory requirements for Class II devices, these being devices for which General Controls alone are insufficient to provide reasonable assurance of the safety and effectiveness of the device, and for which there is sufficient information to establish Special Controls to provide such an assurance.^43^ Special Controls are usually device-specific and include performance standards, post-market surveillance, patient registries, special labeling requirements, premarket data requirements, and operational guidelines. Class III devices are those intended to be used in supporting or sustaining human life or preventing impairment of human health; or that may present a potentially unreasonable risk of illness or injury for which General Controls and Special Controls are insufficient to provide reasonable assurance of the safety and effectiveness of a device; or for which there is insufficient information to make such a determination.^44^ These devices are subject to General Controls and will require PMA. Table 1 provides an overview of the three classes of devices, their risk classification and associated level of regulatory control, and an indication of whether clinical trials are required for market approval.

**Table 1:** Classification of Medical Devices by Risks in the USA, China, and the EU

**USA**	**China**	**EU**
Class I: Low risk of illness or injury Example: Gauze; Adhesive bandages; Toothbrush	Class I: Low risk Example: Simple devices that can be effectively monitored through regular administration	Class I: Low risk Example: Sterile dressings, gloves
Class II: Moderate risk Example: Suture, Diagnostic X-rays	Class II: Moderate risk Example: Nursing device, Reusable surgical device, certain contraceptives and family planning device	Class IIa: Low–medium risk Example: surgical blades, suction equipment Class IIb: Medium–high risk Example: Ventilators, some implants, radiotherapy equipment
Class III: High risk Example: Pacemakers, implantable defibrillators, spinal cord stimulators	Class III: High risk Example: Devices used for life support or sustenance, complex implants	Class III: High-risk Example: Drug-eluting cardiac stents, pacemakers, implantable defibrillators

### III.B. China and the EU

In China, there are many similarities in the regulatory goals and approaches between the NMPA and the FDA, since both authorities are tasked to ensure that medical devices are safe and effective for individual users and for the general public, as the case may be.^45^ A risk-based approach is also applied by the NMPA in the classification of medical devices: Class I devices are of low risk in that their safety and effectiveness can be ensured through routine administration; Class II devices are of moderate risk and require certain controls to ensure safety and effectiveness; and Class III devices are of high risk. For a medical device in Class II or Class III, the manufacturer will need to provide evidence that its device is safe and effective for the intended uses.^46^ Such evidence may be in the form of laboratory testing or limited clinical trials, or documents showing that the device has been approved in its country of origin (eg ISO 13485 certification, FDA approval or ‘CE’ mark in Europe).^47^ The European regulatory regime applies a slightly different risk-based formulation to regulate medical devices.^48^ This regime was previously encapsulated in three directives of the European Commission relating to implantable devices,^49^ in vitro diagnostic devices,^50^ and most other medical devices.^51^ An amendment was made in 2018 to include software into the definition of ‘medical device’ as any instrument or tool intended by the manufacturer to be used for human beings for purposes that include diagnosis, prevention, monitoring, treatment, or alleviation of disease.^52^ Changes to the regulatory framework in the EU have recently come into effect, with the Regulation on Medical Devices (MDR; effective from 26 May 2021) and the In Vitro Diagnostic Devices Regulation (IVDR; effective from 26 May 2022) replacing the three aforementioned Directives.^53^

The classification schemes in the EU and in China broadly reflect the classification rules established by the IMDRF,^54^ and are essentially risk-based. Under the MDR in the EU, a medical device may be classified into one of four categories,^55^ based on the intended purposes or uses and their inherent risks.^56^ A Class I medical device is low risk and do not require independent review.^57^ Every (low-risk) device marketed in the EU must bear a *Conformité Européenne* (CE) mark. While the CE mark simply indicates that a device conforms to regulatory requirements, it is often confused as representing quality.^58^ Medical devices that are non-implantable and considered low risk are ‘self-marked’, in that the manufacturer certifies compliance on its own. Medical devices of moderate and high risks may be assigned to Class IIa, IIb, or III, and will require evidence of safety and efficacy.^59^ High-risk devices must be reviewed by an independent certification body (called a Notified Body), which is a private company approved by authorities of the EU member states to issue CE certification for a fee. However, certain general requirements apply to all medical devices in the EU regardless of their class categorization, which include meeting the general safety and performance requirements (eg information to be supplied by the manufacturers under Annex I of the MDR), complying with reporting requirements under the medical device vigilance system, and being CE marked (except custom-made devices and devices intended for clinical investigation as other requirements apply).

Recent regulatory changes in China and in the EU (all effective from 2021 onwards) widen the range of products that are regulated, strengthen requirements for clinical data and traceability of the devices, introduce more rigorous monitoring of certification bodies, and improve transparency by requiring more product information to be placed in the public domain.^60^ Although it has been observed that the definition of ‘medical device’ may be broader in the EU,^61^ the determination of device qualification is mainly centered around intended use across all three jurisdictions. In establishing intended use, regulators may consider labeling, advertising claims, and other statements, although it has been suggested that the EU regulations allow for greater scrutiny of subjective intent, in addition to intended uses specified in submission documents.^62^

## IV. PARTIAL JUSTIFICATION OF RISK-BASED APPROACH IN HRS

As considered above, a classification scheme based on the risks that a medical device presents by its intended purposes (or use) and its inherent risks is the bedrock of medical device regulation in jurisdictions like China, the EU, and the USA, and endorsed by the IMDRF at a global level. Seen through the lens of the HRS, this risk-based approach satisfies all three of its components in enabling patients in particular to benefit from and (through participation in clinical trials) contribute to scientific and technological progress, in allowing researchers and device developers to push the technology forward, and in grounding an enabling environment. However, the story does not end here. This risk-based approach has been and is likely to remain applicable to non-AI medical devices and many AIMDs that have a fixed risk profile, in that their intended purposes (or uses) and inherent risks will not significantly alter over the course of their product lives. Where an AIMD is allowed to adapt to its data environment in order to optimize its performance in real-time through machine (or deep) learning, such a risk classification scheme begins to unravel.

### IV.A. Risk Objectification in Pathways to Market Approval

While risk objectification by ‘force-fitting’ an AIMD into a conventional medical device category has been effective in paving a way forward to market approval for IDx-DR, its ML capability was ‘locked’ and hence not accounted for. The clinical study that supports the approval was conducted under highly controlled conditions where a relatively small group of carefully selected patients have been recruited to test a diagnostic system along narrow usage criteria at primary care clinics until it was autodidactic.^63^ Hence FDA approval has only been based on IDx-DR functioning like a standard non-AI medical device since the autodidactic functionality has been locked, which in turn rendered the variability of the range of outputs manageable.^64^ At that stage, IDx-DR was not capable of evaluating the most severe forms of diabetic retinopathy that requires urgent ophthalmic intervention.

The version of IDx-DR that was approved by the FDA in 2018 has several components: (1) Fundus camera attached to a computer to take fundus images of a user’s eyes; (2) IDx-DR Client software installed in the computer for users to identify fundus images per eye; (3) IDx-Service software installed on a server hosted at a secure data center to receive fundus images from users; and (4) IDx-DR Analysis, which runs inside IDx-Service and processes the fundus images and returns information on the presence or absence of more-than-mild diabetic retinopathy to IDx-DR Client via IDx-Service.^65^ Collectively, IDx-DR is categorized as a medical device, and was reviewed under the FDA’s de novo premarket review pathway. It was granted Breakthrough Device designation as a medical device that is novel and of low-to-moderate risk.^66^ This PMA pathway was introduced by the US Congress in 1997 for new categories of devices that are not of high risk if they meet two criteria: (1) the new device presents low-to-moderate risk and is likely to meet the statutory standards for classification into Class I or Class II; and (2) the risks and benefits of the new device are sufficiently understood, such that all risks can be effectively mitigated through the application of statutorily prescribed controls.^67^

Unlike IDx-DR, most Class II medical devices in the USA undergo the 510(k) submission process, which requires a substantial equivalence (SE) analysis based on a device previously cleared by the FDA, also referred to as the predicate device.^68^ The device under 510(k) review and its predicate device must have the same intended use and technological characteristics. In the absence of a predicate device, the medical device under review will automatically be classified as Class III and will require a PMA submission. The de novo review pathway was created to allow earlier access to novel medical devices that lack an SE predicate device, as it is not as rigorous as the PMA pathway. Although largely similar to the 510(k) submission process, clinical trials data are usually not required for de novo submissions, but the manufacturer must prove that the risks presented by the device is moderate. In contrast, a manufacturer making a 510(k) submission must show that the risk presented by the device is no greater than its SE device. Given the complexity of a de novo submission, the submission fee is substantially higher than a 510(k) submission. Following regulatory approval of the de novo submission of a novel medical device, a new low-risk (Class I) or moderate-risk (Class II) classification for this device type is created.^69^ Future devices within this device type can be cleared through the 510(k) pathway using the novel device (eg IDx-DR) as a ‘predicate’, usually without the need for clinical evidence to be generated. It is important to recognize that the 510(k) pathway is not foolproof, and ‘fresh’ safety concerns may arise with the recall of the predicate for which the novel medical device was cleared. For instance, a recent study reports that devices were six times more likely to be recalled than similar devices if approved based on comparison with a device that had previously undergone a Class I recall.^70^

The risk profile of IDx-DR was determined based on the results of a prospective clinical trial that obtained retinal fundus photographs and Ocular Coherence Tomography from 900 diabetic patients who were not diagnosed with diabetic retinopathy. Trial results show that IDx-DR was able to achieve sufficient performance when compared with the highest quality reference standard as determined by the Fundus Photography Reading Center, and met predetermined sensitivity and specificity standards for autonomous detection of more-than-mild diabetic retinopathy or diabetic macular edema in people with diabetes and no history of diabetic retinopathy in primary care settings. Approval was granted on the basis that probable benefits of IDx-DR outweighed the probable risks, since early detection of diabetic retinopathy enables early treatment that is needed to prevent significant loss of vision. FDA also notes that the high accuracy of IDx-DR makes the potential risk of false negative low. Even if a false negative should arise, the risk of harm with delayed diagnosis is mitigated by the healthcare provider’s recommendation for follow-up screenings, and that the disease itself progresses slowly. A false positive can also arise but no significant risk is introduced for the patient since examination by an eye care professional occurs regularly.^71^

At the initial stages at least, the regulatory approach that applied to IDx-DR was essentially based on a relatively static conception of risk that enables the continuing application of an essentially point-in-time regulatory scrutiny.^72^ By this approach, regulatory attention and effort are invested mainly in the stages prior to approval being granted, with the expectation that ongoing scrutiny will be relatively less onerous.

### IV.B. Limits of Risk Objectification and ‘Objective’ Control

The regulatory construction of IDx-DR as a ‘risk object’ is accomplished by linking the causal attributes of economic and social risks (especially risks to human safety and agency) to its constitutive algorithms reified as a medical device.^73^ In other words, this ‘risk object’ is made epistemically ‘real’ when embedded in a risk discourse. From the 1970s onwards, a shift in policy in the USA and in Western Europe away from absolute safety in regulatory efforts led to a focus on risk analysis for the determination of risk significance,^74^ as fresh uncertainties were introduced into what was considered to be settled science (or ‘trans-science’, where uncertainties gained prominence and importance).^75^ Risk analysis itself is regarded as comprising at least two distinct parts: risk assessment (where scientific knowledge is relied upon to provide insights on the extent and nature of safety risk), and risk management (where determinations are made as to how risk is to be handled in practice). The origins of the risk assessment–risk management distinction were intended to keep the already well-established basis of risk assessment apart from practice-based (and often values-laden) risk management. As a policy concern, the conceptual insulation of the more science-based part of risk analysis as risk assessment is considered to be advantageous as it ‘*should be* protected from pressures to shift the assessment of risk because the results are politically inconvenient’.^76^

This risk assessment–risk management distinction was formally endorsed by US federal agencies in a report on risk management (called the ‘Red Book’) of the National Research Council (NRC).^77^ Primarily focused on risk to health presented by toxic substances such as asbestos, risk assessment was taken to be concerned with the characterization of the potential adverse health effects of human exposures to environmental hazards. A recommendation in the Red Book was for uniform inference guidelines to be developed to ensure that risk assessments are consistently applied by federal agencies and protected from inappropriate policy influences. In other words, scientific integrity was secured by ensuring that specific economic and social considerations did not have undue influence, even though the risk assessment process was not confined to exclusively scientific considerations. This essentially technical approach to risk assessment broadly reflects popular understanding of risk as a calculative reasoning, or a ‘formal rationality’ that enables the efficient ordering and resolution of problems through technical rules and procedures in structures of economy, society, and state.^78^ By this ordering, risk becomes an instrument of control by directing conduct based on classificatory schemes (risk categorization of medical devices being an example on point) that are aimed at particular goals (of safety and effectiveness levels, for instance).

However, IDx-DR is capable of ML, which is a subset of AI and refers to a set of methods that has the ability to detect patterns in data automatically in order to predict future data trends or for decision-making under uncertain conditions.^79^ Beyond image analysis, AI-based non-image analytical tools may profoundly impact medical practice in a number of ways, including their potential to improve workflow through precision scheduling, identifying patients who are likely to miss appointments, and producing individually customized examination protocols.^80^ In the foreseeable future, the ML feature of medical devices will test the limits of conventional means of regulating AIMDs. The challenges to the binary of risk assessment and risk management will be amplified as such medical devices can rapidly deviate from their intended uses and become less predictable,^81^ and challenge the conventional distinction that is drawn between the research and clinical contexts.^82^ As Preeti Mehrotra and others explain,^83^ the current regulatory approval process is too binary to address the complexities that arise from the interaction between AIMDs and downstream uses that may in turn be governed by fragmented and uncoordinated entities that include hospital policymakers, medical associations, and other stakeholders. The gap between the safety and efficacy profile of an AIMD may be exacerbated by its less tangible form (discreetness), embodiment of more diverse components (discreteness), greater dispersion across geographical and jurisdictional spaces (diffuseness), and variable degrees of explainability.^84^

### IV.C. Falling Short of the HRS

It was in anticipation of these challenges that a new category of ‘risk objects’ known as ‘software as medical device’ (or SaMD) was developed by the IMDRF to govern AIMDs like IDx-DR. If regulators have been insistent that AIMDs should be no different from conventional medical devices in terms of their risk profiles and functional predictability in order to be granted market access approval, the existing risk categorization approach discussed above would have sufficed. However, such a position would have been contrary to the goals of HRS, at least in terms of its three main components considered above. First, patients will not have the opportunity to benefit from, as well as contribute to, the capability of AIMDs to optimize its performance through deeper connectivity with their digital environments in ways that could improve effectiveness and personalize healthcare delivery. Second, scientists will not be able to push forward AI science and technology as the development of AIMDs cannot be limited to experimental trials that are conducted only under strictly controlled conditions. Third, regulators would have failed to provide an enabling regulatory environment that could appropriately balance public interests in facilitating the development of AIMDs, as well as access to AIMDs that are considered safe enough for implementation.

In the section that follows, I consider how the emerging regulatory approach that is centered on SaMD has a broader and more open-ended conception of ‘risks’, and thereby better reflects the goals of HRS. The regulatory character has become more open in terms of the range of possible outcomes that may be accommodated, participatory in relation to the relationship between the regulator and AIMD developers and/or manufacturers, and anticipatory in its response to foreseeable risks. For instance, the FDA provides intensive guidance to developers/manufacturers for efficient device development by collaboratively considering means to expedite evidence generation on more effective treatment or diagnosis of a life-threatening or irreversibly debilitating disease or condition that does not have any approved or cleared alternatives. Following this, I focus on the anticipatory feature of this regulatory approach, which in turn draws on different knowledge domains to constitute and manage AIMDs as a ‘risk object’. Regulatory control is not construed solely in terms of whether the regulated entity behaves strictly in adherence to specific commands, but rather in rendering a degree of predictability to its actions. Crucially, a rights-based approach better accounts for the increasing recognition that risks associated with the actual use of AIMDs cannot be adequately addressed by a set of technical practices, but will require the participation of stakeholders beyond the regulator and developers/manufacturers.

## V. ‘SOFTWARE AS A MEDICAL DEVICE’ AS A NEW REGULATED ENTITY

The IMDRF categorizes AIMDs like IDx-DR as SaMD, which it defines as ‘software intended to be used for one or more medical purposes that perform these purposes without being part of a hardware medical device.’^85^ In a succession of guidance documents, IMDRF recognizes the complex clinical use environment that SaMD may be applied within and can raise or lower the potential to create situations hazardous to patients. Conditions that contribute to the complexity ranges from those that relate to the patient (eg disease progression), those arising from clinical care (eg clinical model applied to derive the output information and the level of clinical evidence available), and those that arise from the operation of the device (eg technological characteristics of the platform the software is intended to operate on), among others. Adopting an essentially consequentialist approach that is focused on intended use of SaMD to achieve particular clinical outcomes, risk characterization is abstracted to two factors:^86^ (1) significance of the information provided by the SaMD to the healthcare decision; and (2) state of the healthcare situation or condition. Important considerations that do not influence the determination of the risk category of the SaMD (eg transparency of the inputs used, technological characteristics used by the SaMD, and more upstream considerations) are not included in this risk categorization framework. It bears repetition and emphasis that by this approach, risk characterization is derived mainly from ‘objective’ information that is provided by the manufacturer on intended use of the SaMD in clinical care. Such use may be significant in one of three ways: (i) to treat or to diagnose; (ii) to drive clinical management; or (iii) to inform clinical management. The significance of an intended use is then associated with a healthcare situation or condition, which may be one of three states: (1) critical situation or condition; (2) serious situation or condition; and (3) non-serious situation or condition. Schematically, Table 2 presents the risk characterization framework comprising four different levels of risk based on the degree of impact on the health of patients or target populations.^87^ As IMDRF also explains, every SaMD will have its own risk category according to its definition statement, even when it is interfaced with other SaMD, other hardware medical devices, or used as a module in a larger system.^88^ Risk categorization thereby relies on ‘accurate and complete’ SaMD definition statement from the manufacturer in the determination of the risk category by associating the significance of the information provided by the SaMD with the healthcare decision and the healthcare situation or condition.^89^ These categories are in relative significance to each other, with the highest category being applicable to SaMD used across multiple healthcare situations or conditions.

**Table 2:** Risk Characterization Framework for Software as a Medical Device

State of Healthcare situation or condition	Significance of information provided by SaMD to healthcare decision		
	Treat or diagnose	Drive clinical management	Inform clinical management
Critical	IV	III	II
Serious	III	II	I
Non-serious	II	I	I

The significance of an intended use is then associated with a healthcare situation or condition, which may be one of three states:

(a) Critical situation or condition, where the type of disease or condition is (i) life-threatening, requires major therapeutic interventions or time critical; (ii) applies to a vulnerable population; and (iii) intended for specialized trained users;

(b) Serious situation or condition, where the type of disease or condition is (i) moderate in progression (and often curable), does not require major therapeutic interventions, and is not time critical; (ii) applies to a target population the is not vulnerable; and (iii) may be used by specialized trained users or lay users; and

(c) Non-serious situation or condition, where the type of disease or condition is (i) slow with predictable progression of disease state, can be managed effectively even if not curable, and requires only minor and normally non-invasive therapeutic interventions.

Level IV (eg SaMD that performs diagnostic image analysis for making treatment decisions in patients with acute stroke, or screens for mutable pandemic outbreak that can be highly communicable through direct contact or other means) being of the highest impact, while Level I (eg SaMD that analyzes optical images to guide next diagnostic action of astigmatism) being the lowest.

This definition of SaMD has been adopted by all major AIMD jurisdictions, along with characteristics that delineate an SaMD and the regulatory implications that follow. In the USA, the FDA has set out an assessment rubric to help manufacturers determine if a device is an SaMD or not.^90^ Similarly, the NMPA in China specified eight special requirements that must be met in addition to those specified as good manufacturing practice for medical devices in general in order for a device to qualify as SaMD.^91^ These requirements include specifications pertaining to design development, quality management, and adverse event monitoring and analysis, and are based on the regulatory principles of the IMDRF and the international standard IEC 62304 (on software),^92^ which the USA and the EU have harmonized their standards with. In the EU, I have earlier on indicated that the MDR adopts a wider definition of medical device to include software and a new classification scheme for software has come into operation.^93^ Innovative regulatory changes accompanied the introduction of SaMD as a new regulated entity, of which I focus on three that are most pertinent to this paper.

### V.A. Responsiveness to Change over Total Product Lifecycle

The regulatory principles proposed by the IMDRF, which have been endorsed by the FDA and similar regulatory authorities in the EU and in China, continue to apply a binary scheme of risk assessment and risk management. However, the divide between this binary is blurring, particularly in view of the fact that the regulatory approach that is put forward emphasizes total product/device lifecycle. This better accounts for modifications that will be made to the device through real-world learning and adaptation. Such adaptation through ML enables a device to change its behavior over time based on new data and optimize its performance in real-time with the goal of improving health outcomes.

Importantly, manufacturers are expected to have an appropriate level of control to manage changes during the lifecycle of the SaMD. IMDRF labels any modifications made throughout the lifecycle of the SaMD, including its maintenance phase, as ‘SaMD Changes’.^94^ Software maintenance is in turn defined in terms of four types of post-marketing modifications that could occur in the software lifecycle processes identified by the International Organization for Standardization,^95^ namely,^96^ (i) adaptive maintenance (modification performed to keep the software product usable in a changed or changing environment); (ii) perfective maintenance (modification to detect and correct latent faults in the software product before they are manifested as failures); (iii) corrective maintenance (reactive modification of a software product performed after delivery to correct discovered problems); and (iv) preventive maintenance (modification of a software product after delivery to detect and correct latent faults in the software product before they become operational faults). When a manufacturer makes changes to SaMD that results in the change of the definition statement, the categorization of SaMD will need to be re-evaluated. As the IMDRF explains, change is inevitable since failures that arise may be due to errors, ambiguities, oversights or misinterpretation of the specification that the software is intended to satisfy, problems in writing code, inadequate testing, incorrect or unexpected usage of the software, or other unforeseen problems. For a software change management process to ensure that the modified SaMD remains safe and of acceptable quality and performance, it will need to include considerations relating to the following:^97^

(1) The socio-technical environment, or the SaMD’s setting of use, typically characterized into spatial (eg locational), activity (eg workflows), social (eg responsibility), technological (eg devices, systems, data sources, and connections), and physical (eg ambient conditions) components;
(2) The technology and system environment, or the ecosystem where the SaMD operates, including installed systems, interconnections, and hardware platforms; and
(3) Information security, which broadly relates to the preservation of confidentiality, integrity, and availability of information.

As indicated above, FDA produced an action plan to develop and apply innovative approaches to the regulation of medical device software and other digital health technologies.^98^ A proposed premarket review mechanism seeks to introduce a Predetermined Change Control Plan (PCCP) in the premarket submission, in order to give effect to the risk categorization and risk management principles, as well as the total product lifecycle approach, of the IMDRF.^99^ The plan will need to include an indication of what anticipated modifications a manufacturer intends to make through learning (or SaMD pre-specifications, or ‘SPS’), and associated methodology that is used to implement the changes in a controlled manner while allowing risks to patients to be managed (referred to as Algorithm Change Protocol). In essence, the proposed changes will place on manufacturers a greater responsibility of monitoring the real-world performance of their medical devices and to make available the performance data through periodic updates on what changes were made as part of the approved SPS and the Algorithm Change Protocol. In totality, these proposed changes will enable the FDA to evaluate and monitor, collaboratively with manufacturers, an ML software as medical device from its premarket development to post-market performance. The nature of the FDA’s regulatory oversight will also become more iterative and responsive in assessing the impact of device optimization on patient safety. In October 2021, a joint statement was issued by the FDA, Health Canada, and the UK’s Medicines and Healthcare products Regulatory Agency on 10 guiding principles for Good Machine Learning Practice (GMLP). These principles include the recommendation of leveraging on multidisciplinary expertise throughout the total product life cycle of an AIMD.^100^

As earlier considered, the regulation of AIMDs as SaMD represents an important regulatory innovation in response to a regulated product that is constantly changing. However, as Kerstin Vokinger and her colleagues explain,^101^ this strategy incorporates significant evidence of safety and effectiveness prior to market entry, preapprove updates and changes that require only minimal regulatory oversight, and a high level of transparency to empower patients and healthcare providers. However, it also renders understanding the performance of post-approval AIMDs and their availability to (as well as impact on) users more challenging.^102^ In the next section, I consider how implementing the HRS could help address these challenges in the regulatory governance of AIMDs.

### V.B. Traceability in Strengthened Post-Market Surveillance

It is generally recognized that testing of software is not sufficient to ensure safety in its operation. Safety features need to be built into the software at the design and development stages, and supported by quality management and post-marketing surveillance after the SaMD has been installed. Post-market surveillance include monitoring, measurement, and analysis of quality data through logging and tracking of complaints, clearing technical issues, determining problem causes and actions to address, identify, collect, analyze, and report on critical quality characteristics of products developed. However, monitoring software quality alone does not guarantee that the objectives for a process are being achieved.^103^ As a quality management system (QMS) requirement, the IMDRF states that maintenance activities should preserve the integrity of the SaMD without introducing new safety, effectiveness, performance, and security hazards. It recommends that risk assessment, including considerations in relation to patient safety and clinical environment and technology and systems environment, should be performed to determine if the changes affect the SaMD categorization and the core functionality of SaMD as set out in its definition statement.^104^ Principles that underscore a QMS are set out as (1) an organizational support structure to provide leadership, accountability, and governance with adequate resources to assure the safety, effectiveness, and performance of the device;^105^ (2) lifecycle support processes that are scalable for the size of the organization and applied consistently across all realization and use processes (ie requirements management, design, development, verification and validation, deployment, maintenance, and decommissioning of the product);^106^ and (3) a set of realization and use processes that are scalable for the type of device and the size of the organization that take into account important elements required for assuring safety, effectiveness, and performance.^107^ These principles are largely reflected in the more collaborative relationship between regulators and the developers and/or manufacturers of AIMDs, but do not currently extend to patients and healthcare providers, as users.

In order to advance ML and to account for a wider range of actual uses that AIMDs could be applied toward, regulatory governance will need to be more participatory in actively and meaningfully involving patients, healthcare providers, and other users or operators. The development of a tracking system for high-risk devices, which is based on the principles set out by the IMDRF on the establishment of a ‘Unique Device Identification’ (UDI), points us in this direction.^108^ Such a system of identification requires these devices to bear a unique identifier in human and machine-readable form in order to improve monitoring and tracking of these devices from the point of their manufacture to their use and eventual decommissioning. In its action plan of January 2021, the FDA acknowledged the need to support a patient-centered approach in initiatives like labeling of medical devices to promote transparency to AIMD users, to support regulatory science efforts to develop methods for evaluating and improving AIMDs, and to advance real-world performance pilots.^109^ In the EU, a UDI system has been introduced pursuant to Article 27 of the MDR, and Article 24 of the IVDR.^110^ In China, a UDI system has similar been introduced pursuant to Article 38 of Order 739 and is piloted in a number of cities and provinces.^111^

### V.C. Increased Transparency

Apart from initiatives like the UDI systems, regulators in these major AIMD jurisdictions also seek to enhance transparency by ensuring greater availability of information to patients, as well as in the public domain. In the EU, an implant card and information about an implantable device must be supplied to the patient in accordance with Article 18 of the MDR.^112^ As a wider initiative, a comprehensive EU database (EUDAMED) on medical devices and certain post-marketing events (such as modifications or withdrawal of products) has been introduced. However, the EUDAMED database is currently only accessible by national competent authorities although a new version of the platform is being developed to serve multiple purposes that include product registration, collaborative arrangements with manufacturers, notification provision, and public dissemination of information.^113^

In a review of the current state of the art in AIMDs, Eric Topol highlights the need for rigorous studies, publication of the results in peer-reviewed journals, and clinical validation in a real-world environment before these devices may be scaled up on implementation and impact on patient care.^114^ Even with the innovative regulatory developments considered above, a number of challenges remain.^115^ These include lack of clarity over the evidential threshold that is needed to meet clinical (for medical devices) or performance (for in vitro devices) evaluation, extent of data that are required for verification and validation, degree to which ML needs to fit with risk management elements of harmonized standards set out by the International Organization of Standards, the need for ML to be human interpretable, and the appropriate level of responsiveness to dynamic changes from ML. To meet these challenges, there will be growing reliance on the participation of patients, healthcare providers, and caregivers in real-world conditions as existing AIMDs apply ML capabilities, even as a greater number of diverse AIMDs are introduced into clinical and ambulatory care. The erstwhile dominant risk-based discourse applied in the regulation of medical devices is silent on participation, beyond established dealings between regulatory agencies and manufacturers (and their representatives). A rights-based approach that is centered on the HRS can help to address this deficiency and thereby steer regulatory and technological innovation forward.

## VI. PARTICIPATION IN REGULATION GOVERNANCE UNDER HRS

Conventionally, the responsibilities of risk assessment and risk management have been largely confined to regulators and device manufacturers or their representatives (known as marketing authorization holders in some jurisdictions). In the earlier sections, I have highlighted how the regulatory character and approach toward AIMDs have started to shift *from* being essentially technical in focus and point-in-time in regulatory intervention *to* being more collaborative, participatory, and anticipatory. In this respect, a participatory governance across society (社会共治) is an explicit regulatory principle in China.^116^ Not much is said about these emerging characteristics, apart from a commitment on the part of regulators to be more engaged and patient-centered. The HRS can help to steer future development in the regulatory governance of AIMDs and SaMDs, and in ways that could meaningfully involve other interested stakeholders, particularly patients with unmet medical needs. For instance, HRS scholars have explained how HRS makes clear state obligations to enable and promote access by children with HIV (a neglected population) to child-specific and child-friendly HIV treatment options through measures that include the adoption of legal and policy frameworks to support research and development that involves children, and in ensuring the participation of marginalized communities in decision-making processes.^117^ There is however no reason why participation should be limited to patients. As noted earlier, ‘benefits’ in HRS include the role of science in forming critical and responsible citizens who are able to participate fully in a democratic society.^118^ In this connection, HRS scholars have also proposed a framework,^119^ as well as indicators,^120^ as a means of evaluating policy and regulatory choices against the obligations derived from the HRS. In this section, I will look into how the HRS could help realize a form of collaboration and participation through regulatory networks that apply a common fund of knowledges.

### VI.A. Regulatory Networks

There is a diverse range of modalities through which participatory engagement and collaborative learning can assume. While each modality has its own goals, character, strengths, and limitations, they generally need to be enabled/supported through regulatory/policy-based platforms or interventions. In view of the greater connectivity of AIMDs, HRS imposes an obligation on states (as regulators) to devise means to enable, as well as sustain, meaningful participation and collaboration. For patients and healthcare providers, meaningful participation could mean access to AIMDs, perhaps even at the design stage, if such access presents the prospect of more independent functioning for patients, and the opportunity to ‘participate in scientific life’.^121^ For regulators and AIMD developers and/or manufacturers, implementing HRS should increase community engagement and participation.^122^

One modality that may be considered is that of a regulatory network, which adapts David Levi-Faur’s definition of a ‘network’ as encompassing a set of relationships of a non-hierarchical and interdependent nature that link a variety of actors.^123^ The network is ‘regulatory’ in the participatory involvement of national regulatory agencies (eg FDA) and supranational entities (eg IMDRF, which could itself be construed as a regulatory network).^124^ The relationships within such a network may be stabilized by institutional and/or network specific arrangements, associations and mechanisms that are directed at achieving particular aims and objectives. From Annelise Riles’s ethnographic research, we know that these goals need not necessarily be premised on a set of shared values, interests, or culture.^125^ In another study, the ‘publics’ of a particular regenerative medicine technology were found to have been co-produced through an institutionalized ‘bioethics-as-public-policy’ national platform.^126^ These ‘publics’—comprised of institutions and institutionally bound individuals—resemble a network in a number of ways. They were brought into a particular set of relationship within a deliberative space created in the main by consultation papers and reinforced through a variety of means that included public meetings, conferences, and feedback sessions. But these ‘networks’ are not static. They vary with, but also shape, the broader discourse of science and expectations as to how science ought to be valued. Arguably, what has become known as a ‘regulatory sandbox’ is yet another modality of network-based participatory engagement.^127^

HRS imposes an obligation on regulators to establish inclusive networks that enable wide participation in the development and governance of AIMDs. Given that the private sector has had a dominant presence in defining the technological agenda and trajectory, whether its Google’s DeepMind in the USA or SenseTime in China, there is arguably a greater need for regulators to spearhead collaboration and participation of the wider public. While commercial interests are not in and of themselves problematic, narrow (or ‘light-touch’) regulatory focus and absence of broader and meaningful participation could be. Clearly, one should be careful to pay heed to the extent to which the agendas for risk assessment are determined mainly by sectorial interest groups that are not incentivized to pursue questions relating to who has the legitimate power to decide which risks should be prioritized and how issues of ‘social benefit’ and ‘technological progress’ should be defined. This in turn presents question of priority and transparency in the public domain, particularly the need to consider what has been left out, notably the ‘social’ and the quality of professional relationships. In this respect, Joshua Kroll reminds us that the ‘black-box’ or opacity concern associated with ML is an unhelpful distraction from thinking about how software systems are validated and how that validation is verified by end users or governance bodies. As he explains:^128^ ‘Opacity in sociotechnical systems results from power dynamics between actors that exist independent of the technical tools in use.’

### VI.B. Common Fund of Knowledges as Anticipatory Knowledge

I explained above that HRS requires public interventions like regulatory networks to enable the right of an interested person to participate in and to enjoy the benefits of scientific progress and its application. The HRS also has an instrumental value, as regulatory networks that give it expression could be an essential tool for the realization of other ESCR, of which the right to health is especially relevant to the present discussion.^129^ In order to be effective in linking the HRS and the right to health, regulatory networks must enable and sustain a culture of learning and experimentation that is, from an epistemic standpoint, pluralistic. In this sense, regulatory networks may be ‘legal’ in form (and to some degree, function), but its epistemic character could be conceptualized as ‘common fund of knowledges’. Mariana Valverde and others illustrate how legal processes are inherent to (but not merely the means of) understanding the risk of a repeat sexual offence under ‘Megan’s Law’, which encompasses the US community notification statutes relating to sexual offenders.^130^ Comprising three tiers, this risk assessment process determines the scope of community notification. In examining the constitutional basis of Megan’s Law, they observe that ‘the courts have emphasized the scientific expertise that is said to be behind the registrant risk assessment scale (RRAS) in order to argue that Megan’s Law is not a tool of punishment but rather an objective measure to regulate a social problem.’ Reliance on Megan’s Law as grounded in objective scientific knowledge has given rise to an ‘intermediary knowledge in which legal actors – prosecutors and judges – are said not only to be more fair but even more reliable and accurate in determining a registrant’s risk of re-offence.’^131^ Instructively, the study illustrates how scientific knowledge may be pooled with other ‘knowledges’ of law and of social care as a ‘common fund’, without having to expand ‘law’ in a doctrinal sense. In this way, legal and regulatory processes may be cognitively and normatively open, in that the RRAS was part of judicial decision-making, but was not itself a legal process. It is in this sense that the epistemic character of regulatory networks should be pluralistic; in comprising a ‘common fund of knowledges’, and also in terms of epistemic commingling, as network actors must work across legal and extra-legal knowledge systems, different fields of law,^132^ as well as governance systems.^133^

It is important to recognize that in giving expression to the HRS in this manner, an essentially static and technical conception of ‘risks’ gives way to one that is anticipatory and collaborative. A ‘common fund of knowledges’ constitutes ‘risks’ as a means of generating ‘anticipatory knowledge’, which may be understood as ‘social mechanisms and institutional capacities involved in producing, disseminating, and using such forms [as] … forecasts, models, scenarios, foresight exercises, threat assessments, and narratives about possible technological and societal futures’.^134^ Like Ian Hacking’s ‘looping effect’,^135^ anticipatory knowledge is about knowledge-making about the future, and could operate as a means to gap-filling. Here, HRS requires that network actors are capacitated with the right to benefit from scientific knowledge and scientific modes of inquiry (eg based on a degree of disinterestedness and organized scepticism), not only as a basis of civic engagement or participation, but also in making decision or policy choices. Since the HRS is linked to and dependent on other human rights (and vice versa), a broader range of normative resources become available. Notably, the ‘common fund of knowledges’ that underscores anticipatory action in international humanitarian law may provide helpful guidance on how affected individuals and communities can be engaged in decision-making ahead of a serious concern or health crisis.^136^ While it is beyond the scope of this paper to discuss how principles and approaches in international humanitarian law could be applied to AIMDs, the points to emphasize here is that the HRS shows how different knowledge systems could be drawn together, often if not always through a mode of collaborative and/or participatory engagement,^137^ in anticipatory knowledge creation and action. Applying a similar analysis, Graeme Laurie and others explain that foresighting as a means of devising anticipatory knowledge is neither simple opinion surveying nor mere public participation.^138^ It must instead be directed at the discovery of shared goals, the development of shared lexicons, the forging of a common vision of the future, and the taking of steps to realize the vision with the understanding that this is being done from a position of partial knowledge about the future. In the context of genomic medicine, Bartha Knoppers explains how networks like the Global Alliance for Genomics and Health similarly pools together a ‘common fund of knowledges’ through interconnectedness across different epistemic and practice domains in the development of an anticipatory governance that enables a learning health system.^139^ Arguably, this approach could better explain the regulatory trajectory for AIMDs (as envisaged by the IMDRF) in terms of the HRS described in the Venice Statement as being more open to beneficial access by, and contributions from, a diverse range of interested parties, and in a manner that promotes the conservation, development, and diffusion of science and technology in the public interest.^140^

While regulatory networks and their epistemic character have instrumental value, HRS as an intrinsic value makes clear their normative commitments. As a rights-based approach, regulatory networks must respect the indivisibility and universality of human rights, and the principle that every person is free and equal in dignity and rights. Participation is already a well-established principle in rights-based approaches, as are the related principles of accountability, non-discrimination and equality, empowerment, and legality, which regulatory networks should also give expression to.^141^ This is consistent with the normative substance of an AIMD regulatory system that scholars have expounded on,^142^ and at a practical level, I have highlighted (in Section V) complementary changes in regulatory mindset and mechanisms, particularly in the shift from traditionally point-in-time to a more holistic total device-specific lifecycle approach.

## VII. CONCLUSION

The regulatory governance of medical devices has conventionally been underpinned by an essentially risk-based discourse, evident in intricate risk classificatory schemes and regulatory mechanisms that operate on the binary of risk assessment and risk management (or safety and effectiveness more generally). For conventional medical devices, this risk-based approach has in turn established particular understandings of regulatory order, but largely confined to regulators and developers/manufacturers interacting at specific points in regulatory time. The key features of risk-based approaches in major medical device jurisdictions like the USA, the EU, and China are considered in Section III. The advent of AIMDs with ML capability, along with policy initiatives like learning healthcare systems, challenges the effectiveness and viability of risk-based approaches, as I explained in Section IV. The high connectivity of AIMDs and the ability to continuously monitor them through a UDI system present novel questions as to how risk objectification and localization should be conceptualized and visualized, among other challenges. Innovative regulatory responses introduced by constituting AIMDs as SaMDs, adopting a total device-specific lifecycle regulatory mindset and other measures considered in Section V, could help to stitch together regulatory lesions that have emerged. However, these measures are only sustainable if there are participatory and collaborative means by which regulators and developers/manufacturers can engage with a wider range of interested stakeholders (particularly AIMD users). In Section VI, I have proposed a rights-based approach that is centered on HRS for the regulatory governance of AIMDs. This approach could be in the form of a regulatory network that draws on a ‘common fund of knowledges’ to formulate anticipatory responses to adaptive AIMDs. To be sure, my proposal is not for the replacement or displacement of risk-based approaches. My point is that the recent measures introduced to sustain risk-based approaches are unlikely to be effective without incorporating rights-based elements or approaches.

In the light that patients and other users will become more involved in the development of AIMDs, through participation in real-world evidence generation programs for instance, safeguards to ensure fairness and equity, privacy and security, and trust are yet to be fully devised and implemented.^143^ HRS lends clarity to the substantive purposes and normative goals that could help advance the regulatory science and art that is applicable to AIMDs, and complements to the more processual focus of the IMDRF’s principles and mechanisms. As I have explained in Section II, HRS imposes an obligation on regulators to ‘adopt legislative, administrative, budgetary, and other measures and establish effective remedies aimed at the full enjoyment of the right to participate in and to enjoy the benefits of scientific progress and its applications.’^144^ Where research is concerned, there are clear ethical and regulatory measures in place to protect people involved in research in most jurisdictions, including the USA, the EU, and China.^145^ The challenge, moving forward, is to devise regulatory approaches that capacitate any interested person to participate and collaborate in shaping sociotechnical imaginations around adaptive AIMDs.

## Footnotes

1 Digital Diagnostics, *History of Digital Diagnostics*, https://www.digitaldiagnostics.com/about/digital-diagnostics/ (accessed Sept. 9, 2023).

2 US Food & Drug Administration, *FDA permits marketing of artificial intelligence-based device to detect certain diabetes-related eye problems, FDA New Release*, 11 April 2018, https://www.fda.gov/newsevents/newsroom/pressannouncements/ucm604357.htm (accessed Sept. 9, 2023).

3 IDx-DR is one of a few fully autonomous AI-based medical devices that has been authorized for use through the de novo process (to be discussed below). Most AI-based medical devices are clinical decision support (CDS) systems, which is defined by the US FDA as ‘a software function that provides healthcare professionals and patients with knowledge and person-specific information, intelligently filtered or presented at appropriate times, to enhance health and healthcare’. See: US Food & Drug Administration, *Step 6: Is the Software Function Intended to Provide Clinical Decision Support?* 14 December 2022, https://www.fda.gov/medical-devices/digital-health-center-excellence/step-6-software-function-intended-provide-clinical-decision-support (accessed Sept. 9, 2023). Most of these CDS systems are not fully autonomous, and their risk profiles are usually comparable with devices previously approved by FDA (known as a predicate device, which will also be discussed later on in this paper). Although uncommon, an autonomous device like IDx-DR best illustrates the limits of the current regulatory approach to medical devices, and why we should consider alternative approaches, including one that is based on the human right to science (HRS) that is proposed in this paper. With technological advancement, CDS software may become increasingly autonomous, although the degree of ‘autonomy’ will depend on a few factors including how such software is embedded in a device, the tasks supported, information provided, and clinicians’ interaction with them. See: D. Lyell, E. Coiera, J. Chen, P. Shah, F. Magrabi, *How machine learning is embedded to support clinician decision making: an analysis of FDA-approved medical devices*, 28 BMJ Health & Care Informatics e100301 (2021).

4 R. McNeil, *Coming to terms with AI*. 26 Eye News, 1, 5 (2019), https://www.eyenews.uk.com/features/ophthalmology/post/coming-to-terms-with-ai (accessed Sept. 9, 2023).

5 China Med Device, *Two AI Software Approved for Diabetic Retinopathy*, 7 September 2020, https://chinameddevice.com/innovation-diabetic-retinopathy/ (accessed Sept. 9, 2023).

6 National Medical Products Administration (formerly known as China Food and Drug Administration), *Main Responsibilities of the National Medical Products Administration*, 18 July 2019, http://english.nmpa.gov.cn/2019-07/18/c_377587.htm (accessed Sept. 9, 2023).

7 US Food & Drug Administration, *How to determine if your Product is a Medical Device*, 29 September 2022, https://www.fda.gov/medicaldevices/deviceregulationandguidance/overview/classifyyourdevice/ucm051512.htm (accessed Sept. 9, 2023).

8 Members of the IMDRF include Australia, Brazil, Canada, China, the European Union, Japan, Russia, Singapore, South Korea, and the USA. See: International Medical Device Regulators Forum, *About IMDRF*, https://www.imdrf.org/about (accessed Sept. 9, 2023).

9 US Food & Drug Administration, *Proposed Regulatory Framework for Modifications to Artificial Intelligence/Machine Learning (AI/ML)-Based Software as a Medical Device (SaMD): Discussion Paper and Request for Feedback*, https://www.fda.gov/files/medical%20devices/published/US-FDA-Artificial-Intelligence-and-Machine-Learning-Discussion-Paper.pdf (accessed Sept. 9, 2023).

10 US Food & Drug Administration, *Artificial Intelligence/Machine Learning (AI/ML)-Based Software as a Medical Device (SaMD) Action Plan*, https://www.fda.gov/media/145022/download (accessed Sept. 9, 2023).

11 C. W. L. Ho, D. Soon, K. Caals, J. Kapur, *Governance of automated image analysis and artificial intelligence analytics in healthcare*, 74 Clinical Radiology 329, 330.

12 Sheila Jasanoff, *Future Imperfect: Science, Technology, and the Imaginations of Modernity*, in Dreamscapes of Modernity: Sociotechnical Imaginaries and the Fabrication of Power 4 (Sheila Jasanoff and Sang-Hyun Kim eds., 2015). As Sheila Jasanoff explains, sociotechnical imaginaries provide an account of why technological trajectories diverge across jurisdictions and periods, and the roles that technologies play in connecting a person’s subjective self-understanding of health and well-being to a shared social or moral order. Analytically, it cuts through the binary of structure and agency, and combines some of the subjective and psychological dimensions of agency with the structural features of technological systems, policy styles, organizational behaviors, and political cultures.

13
Ian Wilkinson, Risk, Vulnerability and Everyday Life 26 (2010).

14 Unlike risk-based approaches, rights-based approaches put the focus on persons and people rather than the technology. In fields like mental health, rights-based approaches are gaining traction. For instance, see: V. Haldane, A-S Jung, C. D. Foo, P. Shrestha, E Urdaneta, E. Turk, et al., *Integrating HIV and substance misuse services: a person-centred approach grounded in human rights*, 9(8) The Lancet Psychiatry 676. See also: World Health Organization, *Comprehensive mental health service networks: Promoting person-centred and rights-based approaches*, 9 June 2021, https://www.who.int/publications/i/item/9789240025844 (accessed Sept. 9, 2023).

15 A promising area of development lies in software that will meaningfully process massive sets of data quickly, accurately, and inexpensively, and for machines that are capable of seeing and doing things that are not humanly possible. With deep-learning methodologies, a healthcare system may become smarter by learning more from electronic health record data, but the active participation of healthcare providers and patients is similarly indispensable. For an illustration of what such a learning healthcare system could look like, see: B. Norgeot, B. S. Glicksberg, A. J. Butte, *A call for deep-learning healthcare*. 25 Nature Medicine 14 (2019).

16 B. Babic, S. Gerke, T. Evgeniou, I. G. Cohen, *Algorithms on regulatory lockdown in medicine: Prioritizing risk monitoring to address the ‘update problem’*, 366 Science 1202 (2019).

17 M. Bidault, *Considering the Right to Enjoy the Benefits of Scientific Progress and Its Applications As a Cultural Right: A Change in Perspective*, in The Right to Science: Then and Now 140–149 (Porsdam and Porsdam Mann, eds., 2021).

18 S. Jasanoff, *supra* note 12, at 24–26.

19
Roger Brownsword, Law, Technology and Society: Re-Imagining the Regulatory Environment (2019).

20
R. Brownsword, E. Scotford, K. Yeung, *Law, Regulation and Technology: The Field, Frame, and Focal Questions*, in The Oxford Handbook of Law, Regulation and Technology (R. Brownsword, E. Scotford and K. Yeung eds., 2017).

21 S. Taylor-Alexander, E. S. Dove, I. Fletcher, A. G. Mitra, C. McMillan, G. Laurie, *Beyond regulatory compression: confronting the liminal spaces of health research regulation*, 8 Law Innovation and Technology 149 (2016).

22 United Nations (General Assembly), Universal declaration of human rights (217 [III]A). Paris (1948), https://www.un.org/en/about-us/universal-declaration-of-human-rights (accessed Sept. 9, 2023).

23 United Nations (General Assembly), International Covenant on Economic, Social, and Cultural Rights, Treaty Series 999 (December): 171 (1966), https://www.refworld.org/docid/3ae6b36c0.html (accessed Sept. 9, 2023). Complaint and inquiry mechanisms are established under the Optional Protocol to the International Covenant on Economic, Social and Cultural Rights, but the discussion in this paper will be limited to the normative content of the HRS. For the Optional Protocol, see: United Nations (General Assembly), Optional Protocol to the International Covenant on Economic, Social and Cultural Rights, A/RES/63/117 10 December 2008, https://www.ohchr.org/en/instruments-mechanisms/instruments/optional-protocol-international-covenant-economic-social-and (accessed Sept. 9, 2023).

24 Committee on Economic, Social and Cultural Rights of the United Nations Economic and Social Council, *General comment No. 25 (2020) on science and economic, social and cultural rights (article 15(1)(b), (2), (3) and (4) of the International Covenant on Economic, Social and Cultural Rights)*, E/C.12/GC25, 30 April 2020, https://tbinternet.ohchr.org/_layouts/15/treatybodyexternal/Download.aspx?symbolno=E%2fC.12%2fGC%2f25&Lang=en (accessed Sept. 9, 2023).

25 R Yotova, B Knoppers, *The Right to Benefit from Science and Its Implications for Genomic Data Sharing*, 31 European journal of International Law 665 (2020); A. Boggio, B. Knoppers, et al., *The Human Right to Science and the Regulation of Human Germline Engineering*, 2 The Crispr Journal 134 (2019).

26 Article 27 of the Universal Declaration of Human Rights is noted to be especially relevant as it articulates the right of all individuals to freely participate in the cultural life of the community, to enjoy the arts and to share in scientific advancement and its benefits. *Supra* n 22, at paragraphs 3 and 10.

27 L. London, H. Cox, F. Coomans, *Multidrug-Resistant TB: Implementing the Right to Health through the Right to Enjoy the Benefits of Scientific Progress*, Health Hum Rights. 2016 Jun; 18(1):25–41, at 28. The United Nations Human Rights Office of the High Commissioner (UNHRC) explains this as ‘an environment that promotes the conservation, development, and diffusion of science and technology, and the freedom indispensable for scientific research’. See UNHRC, The right to benefit from scientific progress and its applications, https://www.ohchr.org/en/special-procedures/sr-cultural-rights/right-benefit-scientific-progress-and-its-applications (accessed Sept. 9, 2023).

28 UNCESCR, *supra* note 24, adapted from paragraph 52.

29 UNCESCR, *id*., at paragraph 51.

30
*Id*., at paragraph 63.

31
*Id*., at paragraphs 67–76. While the UNCESCR’s discussion mainly focuses on medical products like pharmaceutical drugs and vaccines, it is also applicable to medical devices. Those aspects of the discussion that relate to intellectual property is not considered as they fall outside the scope of this paper.

32
*Id*., at paragraph 70.

33
*Id*., at paragraph 67.

34
*Id*., at paragraph 71.

35 21 USC §201(h).

36 Global Harmonization Task Force, *Definitions of the Terms ‘Medical Device’ and ‘In Vitro Diagnostic (IVD) Medical Device’*, GHTF/SG1/N071:2012, 16 May 2012, https://www.imdrf.org/sites/default/files/docs/ghtf/final/sg1/technical-docs/ghtf-sg1-n071-2012-definition-of-terms-120516.pdf (accessed Sept. 9, 2023).

37 World Health Organization, *Medical Devices: WHO Response*, https://www.who.int/health-topics/medical-devices#tab=tab_2 (accessed Sept. 9, 2023).

38 US Food & Drug Administration, *Classification of Products as Drugs and Devices & Additional Product Classification Issues: Guidance for Industry and FDA Staff*, https://www.fda.gov/media/80384/download (accessed Sept. 9, 2023).

39 US Food & Drug Administration, *Device Classification Panels*, 31 August 2018, https://www.fda.gov/medical-devices/classify-your-medical-device/device-classification-panels (accessed Sept. 9, 2023).

40 The class to which a medical device is designated depends on its risk profile, intended use, indications for use, technological characteristics, and the regulatory controls that should be in place for a reasonable assurance of safety and effectiveness. See: US Food & Drug Administration, *Step 3: Pathway to Approval*, 2 September 2018, https://www.fda.gov/patients/device-development-process/step-3-pathway-approval (accessed Sept. 9, 2023).

41 A list of class I devices and class II devices are exempted from premarket notification requirements, although some limitations apply. See: 84 FR 71794, https://www.federalregister.gov/documents/2019/12/30/2019-27394/medical-devices-exemptions-from-premarket-notification-for-class-i-and-class-ii-devices (accessed Sept. 9, 2023). Additionally, exemption may apply for a humanitarian use device. See: US Food & Drug Administration, *Humanitarian Device Exemption*, 3 October 2022, https://www.fda.gov/medical-devices/premarket-submissions-selecting-and-preparing-correct-submission/humanitarian-device-exemption (accessed Sept. 9, 2023).

42 US Food & Drug Administration. *Regulatory Controls*, 27 March 2018, https://www.fda.gov/medical-devices/overview-device-regulation/regulatory-controls (accessed Sept. 9, 2023).

43 US Food & Drug Administration, *Regulatory Controls*, 27 March 2018, https://www.fda.gov/medical-devices/overview-device-regulation/regulatory-controls#special (accessed Sept. 9, 2023). See also: US Food & Drug Administration, *Class II Special Controls Documents*, 14 June 2022, https://www.fda.gov/medical-devices/guidance-documents-medical-devices-and-radiation-emitting-products/class-ii-special-controls-documents (accessed Sept. 9, 2023).

44 US Food & Drug Administration, *Classify Your Medical Device*, 2 July 2020, https://www.fda.gov/medical-devices/overview-device-regulation/classify-your-medical-device (accessed Sept. 9, 2023).

45 State Council of the People’s Republic of China, *Decree No. 739 on Regulations on Supervisory Management of Medical Devices,* 1 June 2021.

46 National Medical Products Administration (China Food and Drug Administration, as it then was), Rules for Classification of Medical Devices (Decree No. 15 of China Food and Drug Administration), issued on 14 July 2015 (updated 11 October 2019), Article 4, http://english.nmpa.gov.cn/2019-10/11/c_415411.htm (accessed Sept. 9, 2023).

47
*Supra* n. 45, Article 15 of Order 739.

48 Paul Quinn suggests that the EU has applied a non-risk-based strategy although it is difficult to see how the overall regime is not ultimately premised on risk classification similar to the regulatory approaches in the USA and China. See Paul Quinn, *The EU commission’s risky choice for a non-risk based strategy on assessment of medical devices*, 33 Computer Law & Security Review 361, 367–8 (2017).

49 Council Directive 90/385/EEC of 20 June 1990 on the approximation of the laws of the Member States relating to active implantable medical devices [1990] OJ L 189.

50 Council Directive 98/79/EC of the European Parliament and the Council of 27 October 1998 on in vitro diagnostic medical devices [1998] OJ L 331/1.

51 Council Directive 93/42/EEC of 14 June 1993 concerning medical devices. OJ L 169. Article 1 of this Directive defines ‘medical device’ as “any instrument, apparatus, appliance, software, material, or other article, whether used alone or in combination, including the software intended by its manufacturer to be used specifically for diagnostic and/or therapeutic purposes and necessary for its proper application, intended by the manufacturer to be used for human beings for the purpose of diagnosis, prevention, monitoring, treatment, or alleviation of disease; diagnosis, monitoring, treatment, alleviation of or compensation for an injury or handicap; investigation, replacement, or modification of the anatomy or of a physiological process; control of contraception; and which does not achieve its principal intended action in or on the human body by pharmacological, immunological, or metabolic means, but which may be assisted in its function by such means.”

52 European Commission. *MDCG 2018–2 Future EU medical device nomenclature – description of requirements*, https://ec.europa.eu/docsroom/documents/28668 (accessed Sept. 9, 2023).

53 Regulation (EU) 2017/745 of the European Parliament and of the Council of 5 April 2017 on medical devices [2017] OJ L 117/1; and Regulation (EU) 2017/746 of the European Parliament and of the Council of 5 April 2017 on in vitro diagnostic medical devices [2017] OJ L 117/176. For an overview of these legal instruments, see: European Medicines Agency, *Medical devices*, https://www.ema.europa.eu/en/human-regulatory/overview/medical-devices#:~:text=They%20are%20regulated%20at%20EU,has%20passed%20a%20conformity%20assessment. (accessed Sept. 9, 2023).

54 Global Harmonization Task Force (now known as IMDRF), *Principles of Medical Devices Classification*, GHTF/SG1/N77:2012, 2 November 2012, https://www.imdrf.org/sites/default/files/docs/ghtf/final/sg1/technical-docs/ghtf-sg1-n77-2012-principles-medical-devices-classification-121102.pdf (accessed Sept. 9, 2023).

55 MDR, *supra* note 53, Article 51 and Annex VIII. The ‘classification rules’ broadly correspond to those set out by the IMDRF.

56 The classification scheme for medical devices in the European Union is similarly risk-based, with focus on the vulnerability of the human body and the potential risks associated with the devices. The risk evaluation criteria includes duration of contact with the body, degree of invasiveness, local as opposed to systemic effect, potential toxicity, the part of the body affected by the use of the device, and whether the device depends on a source of energy. See: Medical Device Coordination Group, *MDCG 2021–24 Guidance on classification of medical devices*, October 2021, https://health.ec.europa.eu/system/files/2021-10/mdcg_2021-24_en_0.pdf (accessed Sept. 9, 2023).

57 Class I devices may be further subdivided into Is—sterile condition, Im—measuring function, and Ir 0 reusable surgical. See: MDR, *supra* note 53, Article 52(7)(a), (b), and (c).

58 G. A. Van Norman. *Drugs and Devices: Comparison of European and U.S. Approval Processes*. 1 JACC Basic to Translational Science 399 (2016).

59 A similar risk classification scheme is applied in the IVDR, whereby an in vitro medical device may be classified into one of four risk classes: class A (low individual risk and low public health risk); class B (moderate individual risk and/or low public health risk); class C (high individual risk and/or moderate public health risk); and class D (high individual risk and high public health risk). See: IVDR, *supra* note 53, Article 47.

60 F. Pesapane, C. Volonte, M. Codari, F. Sardanelli, *Artificial intelligence as a medical device in radiology: ethical and regulatory issues in Europe and the United States*, 9 Insights into Imaging 745 (2018).

61 T. Minssen, S. Gerke, M. Aboy, N. Price, G. Cohen, *Regulatory responses to medical machine learning*, 7(1) Journal of Law and the Biosciences lsaa002 (2020).

62
Johan Ordish, Hannah Murfet, Alison Hall, Algorithms as Medical Devices, 17–18 (2019), https://www.phgfoundation.org/report/algorithms-as-medical-devices (accessed Sept. 9, 2023).

63 M. D. Abràmoff, P. T. Lavin, M. Birch, N. Shah, J. C. Folk, *Pivotal trial of an autonomous AI-based diagnostic system for detection of diabetic retinopathy in primary care offices*, 1 Digital medicine 39 (2018).

64 P. A. Keane, E. J. Topol, *With an eye to AI and autonomous diagnosis*, 1 Digital medicine 40 (2018).

65 US Food & Drug Administration, *De Novo Classification Request for IDx-DR*, https://www.accessdata.fda.gov/cdrh_docs/reviews/DEN180001.pdf (accessed Sept. 9, 2023).

66
*Id*.

67 J. P. Jarow, J. H. Baxley, *Medical devices: US medical device regulation*, 33 Urologic Oncology: Seminars and Original Investigations 128 (2015).

68 US Food & Drug Administration, *The 510(k) Program: Evaluating Substantial Equivalence in Premarket Notifications [510(k)]: Guidance for Industry and Food and Drug Administration Staff*, 28 July 2014, https://www.fda.gov/media/82395/download (accessed Sept. 9, 2023).

69 US Food & Drug Administration, *Section 513(f)(2) – Evaluation of automatic class III designation, guidance for industry and CDRH staff*, https://www.fda.gov/media/72674/download (accessed Sept. 9, 2023).

70 K.T. Kadakia, S.S. Dhruva, C. Caraballo, J.S. Ross, H.M. Krumholz, *Use of Recalled Devices in New Device Authorizations Under the US Food and Drug Administration’s 510(k) Pathway and Risk of Subsequent Recalls*, 329(2) JAMA 136 (2023). See also: A.O. Everhart, S. Sen, A.D. Stern, Y. Zhu, P. Karaca-Mandic, *Association Between Regulatory Submission Characteristics and Recalls of Medical Devices Receiving 510(k) Clearance*, 329(2) JAMA 144 (2023).

71 FDA, *supra* note 2. Since the FDA’s marketing authorization of IDx-DR, there is some evidence to suggest that this AIMD improves patient outcome, but research is still ongoing. See: A. Grzybowski, P. Brona, *Analysis and Comparison of Two Artificial Intelligence Diabetic Retinopathy Screening Algorithms in a Pilot Study: IDx-DR and Retinalyze*, 10(11) Journal of Clinical Medicine 2352 (2021).

72 US Food & Drug Administration, *Digital Health Software Precertification (Pre-Cert) Program*, 26 September 2022, https://www.fda.gov/medical-devices/digital-health/digital-health-software-precertification-pre-cert-program (accessed Sept. 9, 2023).

73 Stephen Hilgartner, *The Social Construction of Risk Objects: or, How to Pry Open Networks of Risk*, in Organizations, Uncertainties, and Risk 39 (J. F. Short and L. Clarke eds. 1992). See also: A. Bowser, M. Sloan, P. Michelucci and E. Pauwels. Artificial Intelligence: A Policy-Oriented Introduction (2017), https://www.scribd.com/document/364545974/Artificial-Intelligence-A-Policy-Oriented-Introduction#download&from_embed (accessed Sept. 9, 2023).

74
Heather E. Douglas, Science, Policy, and the Value-Free Ideal (2009).

75
Sheila Jasanoff, The Ethics of Invention: Technology and the Human Future (2016).

76
*Id*. at 141 (emphasis in original).

77
National Research Council, Risk Assessment in the Federal Government: Managing the Process 18 (1983).

78 Max Weber, *Religious rejections of the world and their directions*, in From Max Weber 324 (H. H. Gerth and C. W. Mills eds.,1948).

79
Kevin P. Murphy, Machine Learning: A Probabilistic Perspective (2012).

80
Eric Topol, Deep Medicine: How Artificial Intelligence Can Make Healthcare Human Again (2019). See also: A. Tang, R. Tam, A. Cadrin-Chênevert, W. Guest, J. Chong, J. Barfett, L. Chepelev, R. Cairns, J.R. Mitchell, M. D. Cicero, M. G. Poudrette, J. L. Jaremko, C. Reinhold, B. Gallix, B. Gray, R. Geis, *Canadian Association of Radiologists White Paper on Artificial Intelligence in Radiology*, 69 Canadian Association of Radiologists Journal 120 (2018).

81 H. Yu, *Regulation of Digital Health Technologies in the European Union: Intended versus Actual Use*, in The Future of Medical Device Regulation: Innovation and Protection 103–114 (I. G. Cohen, T. Minssen, W. Nicholson Price II, C. Robertson and C. Shachar eds., 2022).

82 C. W. L. Ho, *When Learning Is Continuous: Bridging the Research–Therapy Divide in the Regulatory Governance of Artificial Intelligence as Medical Devices*, in The Cambridge Handbook of Health Research Regulation 277–286 (G. Laurie, E. Dove, A. Ganguli-Mitra, C. McMillan, E. Postan, N. Sethi, et al. eds., 2021).

83 P. Mehrotra, D. J. Weber, A. Sarpatwari, *Preventing Medical Device-Borne Outbreaks: The Case of High-Level Disinfection Policy for Duodenoscopes*, in The Future of Medical Device Regulation: Innovation and Protection 192–202 (I. G. Cohen, T. Minssen, W. Nicholson Price II, C. Robertson and C. Shachar eds., 2022).

84 M. U. Scherer, *Regulating artificial intelligence systems: risks, challenges, competencies and strategies*, 29 Harvard Journal of Law & Technology 354 (2016).

85 International Medical Device Regulators Forum, *Software as a Medical Device (SaMD): Key Definitions*, https://www.imdrf.org/documents/software-medical-device-samd-key-definitions (accessed Sept. 9, 2023).

86 International Medical Device Regulators Forum, *‘Software as a Medical Device’: Possible Framework for Risk Categorization and Corresponding Considerations, IMDRF/SaMD WG/N12FINAL:2014*, paragraph 4, http://www.imdrf.org/docs/imdrf/final/technical/imdrf-tech-140918-samd-framework-risk-categorization-141013.pdf (accessed Sept. 9, 2023).

87
*Id*. at 14, paragraph 7.2.

88
*Id*. at 13–14, paragraph 7.1.

89
*Id*.

90 US Food & Drug Administration, *Your Clinical Decision Support Software: Is it a Medical Device?* 27 September 2022, https://www.fda.gov/medical-devices/software-medical-device-samd/your-clinical-decision-support-software-it-medical-device (accessed Sept. 9, 2023).

91 National Medical Products Administration, *Good Manufacturing Practice Annex on Standalone Software, 7 July 2019*, http://www.nmpa.gov.cn/WS04/CL2138/338970.html (document in Chinese, accessed Sept. 9, 2023).

92
International Electrotechnical Commission, Iec 62304 + Amd1:2015 Csv Medical Device Software – Software Life Cycle Processes (2015).

93 MDR, *supra* note 53, Article 2(1) and Section 6.3 of Chapter III of Annex VIII (Rule 11). Broadly speaking, software that monitors physiological processes is classified as class IIa, and a high-risk classification applies if the information that the software provides is used to take decisions that could lead to serious deterioration of a person’s state of health or a surgical intervention (the device would then be in the class IIb category), or could cause death or an irreversible deterioration of a person’s state of health (in which case, the device would be in the class III category).

94 IMDRF, *supra* note 85 at 22–23, paragraph 8.2.

95
International Organization of Standards, ISO/IEC 14764:2006 Software Engineering – Software Life Cycle Processes – Maintenance (2^nd^ ed., 2006).

96 IMDRF, *supra* note 85, at 22.

97
*Id*., at 22–27, paragraph 9.

98 FDA *supra* note 10. As part of an action plan to facilitate the implementation of SaMD, the US FDA indicated that it will introduce a tailored regulatory framework with regulatory specifications on the Predetermined Change Control Plan (PCCP).

99 H Benjamin Harvey, Vrusha Gowda, *How the FDA Regulates AI*, 27 Academic Radiology 58, 59 (2020).

100 US Food & Drug Administration, Health Canada and Medicines & Healthcare products Regulatory Agency (UK), *Good Machine Learning Practice for Medical Device Development: Guiding Principles*, October 2021, https://www.fda.gov/medical-devices/software-medical-device-samd/good-machine-learning-practice-medical-device-development-guiding-principles (accessed Sept. 9, 2023). In the Action Plan of January 2021, the FDA indicated its intent to facilitate the harmonized development of Good Machine Learning Practice (GMLP) by being more involved in collaborative communities and consensus standards development efforts.

101 K. N. Vokinger, T. J. Hwang, A. S. Kesselheim, *Lifecycle Regulation and Evaluation of Artificial Intelligence and Machine Learning-Based Medical Devices*, in The Future of Medical Device Regulation: Innovation and Protection 13–21 (I. G. Cohen, T. Minssen, W. Nicholson Price II, C. Robertson and C. Shachar eds., 2022).

102 M. Herder, N. Cortez, *A ‘DESI’ for Devices? Can a Pharmaceutical Program from the 1960s Improve FDA Oversight of Medical Devices?* in The Future of Medical Device Regulation: Innovation and Protection 129–140 (I. G. Cohen, T. Minssen, W. Nicholson Price II, C. Robertson and C. Shachar eds., 2022).

103 International Medical Device Regulators Forum, *Software as a Medical Device (SaMD): Application of Quality Management System, IMDRF/SaMD WG/N23 FINAL:2015*, para. 7.5 http://www.imdrf.org/docs/imdrf/final/technical/imdrf-tech-151002-samd-qms.pdf (accessed Sept. 9, 2023).

104
*Id*. at 26, paragraph 8.6.

105
*Id*. at 10–11, paragraph 6.

106
*Id*. at 12–18, paragraph 7.

107
*Id*. at 19–28, paragraph 8.

108 International Medical Device Regulators Forum, *Unique Device Identification System (UDI system) Application Guide*, http://www.imdrf.org/docs/imdrf/final/consultations/imdrf-cons-udi-system-n48-180712.pdf (accessed Sept. 9, 2023).

109 FDA, *supra* note 10, at 4–5.

110 MDR & IVDR, *supra* note 53. See also: European Commission, *Unique Device Identification (UDI) System under the EU medical devices Regulations 2017/745 and 2017/746*, 1 August 2020, https://health.ec.europa.eu/system/files/2020-09/md_faq_udi_en_0.pdf (accessed Sept. 9, 2023).

111 State Council of China, *supra* note 45. See also: National Medical Products Administration, *Medical Device Unique Identification System Rules (No. 66 of 2019),* 27 August 2019, https://www.nmpa.gov.cn/directory/web/nmpa/ylqx/ylqxggtg/ylqxqtgg/20190827092601750.html (document in Chinese, accessed Sept. 9, 2023).

112 MDR, *supra* note 53.

113 European Commission, *European Database on Medical Devices* (EUDAMED). https://ec.europa.eu/growth/sectors/medical-devices/new-regulations/eudamed_en (accessed Sept. 9, 2023).

114 E. J. Topol, *High-performance medicine: the convergence of human and artificial intelligence*, 25 Nature Medicine 44, 52 (2019).

115 J. Ordish et al., *supra* note 62 at 25–33.

116 State Council of China, *supra* note 45, Article 5 of Order 739.

117 M. L. Scanlon, G. Macnaughton, C. Sprague, *Neglected Population, Neglected Right: Children Living with HIV and the Right to Science*, 19 Health and Human Rights Journal 169 (2017).

118 UNCESCR, s*upra* n 24, at paragraphs 3 and 10.

119 S. Porsdam Mann, Y. Donders, H Porsdam, *The Right to Science in Practice: A Proposed Test in Four Stages*, in The Right to Science: Then and Now 231–245 (Porsdam and Porsdam Mann, eds., 2021).

120 A Boggio, B. Gran, *A Proposal for Indicators of the Human Right to Science*, in The Right to Science: Then and Now 268–285 (Porsdam and Porsdam Mann, eds., 2021).

121 Bidault, *supra* note 17.

122 See for instance: V. J. Bradley, *Implications of the Right to Science for People with Disabilities*, in The Right to Science: Then and Now 150–165 (Porsdam and Porsdam Mann, eds., 2021). With reference to the Convention on the Rights of People with Disabilities, Bradley explains that the HRS holds the promise of the ability for disable people to live independently, re-examine the construct of disability and increase participation in the community.

123 Levi-Faur, D. (2011), *Regulatory Networks and Regulatory Agencification: Towards a Single European Regulatory Space*. Journal of European Public Policy 18, 810–829.

124 Papadopoulos, Y. (2018), *How does knowledge circulate in a regulatory network? Observing a European Platform of Regulatory Authorities meeting*. Regulation & Governance, 12: 431–450.

125
Annelise Riles, The Network Inside Out 58–9, 68 (2001). In her study of Fijian non-governmental organizations that participated in the 1995 United Nations Beijing Conference on Women, Annelise Riles observes that networkers they understood themselves to be sharing in their involvement in a certain network that was a form of institutionalized association devoted to information sharing. What defined networkers most of all was the fact that they were personally and institutionally connected or knowledgeable about the world of Pacific institutions and networks. It was the work of creating documents, organizing conferences, or producing funding proposals that generated a set of personal relations that drew people together and also created divisions of its own.

126
Calvin Wai Loon Ho, Juridification in Bioethics (2016).

127 Financial Conduct Authority, *Regulatory sandbox lessons learned report*, https://www.fca.org.uk/firms/innovation/regulatory-sandbox (accessed Sept. 9, 2023).

128 J. A. Kroll, *The fallacy of inscrutability*, 376 Philosophical Transactions of the Royal Society A 20180084, 11 (2018).

129 UNCESCR, *supra* note 24, Part D. See also: F. Bustreo, C. F. J. Doebbler, *The Rights-Based Approach to Health*, in Foundations of Global Health & Human Rights 89–110 (L. O. Gostin, B. M. Meier eds., 2020).

130 M. Valverde, R. Levi, D. Moore, *Legal Knowledges of Risk*, in Law and Risk 103 (Law Commission of Canada ed., 2005).

131
*Id*. at 106.

132 J. Meszaros, M. Corrales Compagnucci, T. Minssen. *The Interaction of the Medical Device Regulation and the GDPR: Do European Rules on Privacy and Scientific Research Impair the Safety and Performance of AI Medical Devices?* in The Future of Medical Device Regulation: Innovation and Protection 77–90 (I. G. Cohen, T. Minssen, W. Nicholson Price II, C. Robertson and C. Shachar eds., 2022).

133 UNCESCR explains the need for participation and collaboration at the epistemic level in its emphasis on cooperation across many levels: *supra* note 24, Part VI. See also: C. W. L. Ho, K. Caals, *A Call for an Ethics and Governance Action Plan to Harness the Power of Artificial Intelligence and Digitalization in Nephrology*, 41 Seminars In Nephrology 282 (2021).

134 N. Nelson, A. Geltzer, S. Hilgartner, *Introduction: the anticipatory state: making policy-relevant knowledge about the future*, 35 Science and Public Policy 546, 553 (2008).

135 Ian Hacking, *The looping effects of human kinds*, in Causal Cognition: A Multidisciplinary Debate 351 (D. Sperber, D. Premack, and A. J. Premack eds., 1995).

136 C. Droege, E. Giorgou, *How international humanitarian law develops*, 104 International Review of the Red Cross 1798 (2022).

137 See for instance the growing movement toward mandated social reporting requirements. P. Redmond, *Regulating through reporting: an anticipatory assessment of the Australian Modern Slavery Act*, 26 Australian Journal of Human Rights 5 (2020).

138 G. Laurie, S. H. E. Harmon, F. Arzuaga, *Foresighting Futures: Law, New Technologies, and the Challenges of Regulating for Uncertainty*, 4 Law, Innovation and Technology (2012).

139 B. Knoppers, *The right to benefit from science and data sharing*, 17 Oncology Exchange 29 (2018).

140 American Association for the Advancement of Science, *Venice Statement on the Right to Enjoy the Benefits of Scientific Progress and its application*, 2009, https://www.aaas.org/sites/default/files/VeniceStatement_July2009.pdf (accessed Sept. 9, 2023).

141 United Nations Sustainable Development Group, *The Human Rights Based Approach to Development Cooperation Towards a Common Understanding Among UN Agencies*, September 2003, https://unsdg.un.org/resources/human-rights-based-approach-development-cooperation-towards-common-understanding-among-un (accessed Sept. 9, 2023).

142 C. Cath, S. Wachter, B. Mittelstadt, M. Taddeo, L. Floridi, *Artificial Intelligence and the ‘Good Society’: the US, EU, and UK approach*, 24 Science and Engineering Ethics 505 (2018). See also: High-Level Expert Group on Artificial Intelligence, *Ethics Guidelines for Trustworthy AI*, https://ec.europa.eu/futurium/en/ai-alliance-consultation.1.html (accessed Sept. 9, 2023).

143 Ethical, legal, and regulatory concerns have already been well articulated in important forums. See, for instance, AI100 Standing Committee and Study, *Artificial Intelligence and Life in 2030*, https://ai100.stanford.edu/2016-report (accessed Sept. 9, 2023); Nuffield Council on Bioethics, *Artificial intelligence (AI) in healthcare and research*, http://nuffieldbioethics.org/project/briefing-notes/artificial-intelligence-ai-healthcare-research (accessed Sept. 9, 2023); National Academy of Sciences, *Artificial Intelligence and Machine Learning to Accelerate Translational Research: Proceedings of a Workshop in Brief*, https://www.nap.edu/catalog/25197/artificial-intelligence-and-machine-learning-to-accelerate-translational-research-proceedings (accessed Sept. 9, 2023).

144 UNCESCR, *supra* note 24, paragraph 45.

145
*Id*., paragraph 43.

